# Dysregulation in Akt/mTOR/HIF-1 signaling identified by proteo-transcriptomics of SARS-CoV-2 infected cells

**DOI:** 10.1080/22221751.2020.1799723

**Published:** 2020-07-31

**Authors:** Sofia Appelberg, Soham Gupta, Sara Svensson Akusjärvi, Anoop T. Ambikan, Flora Mikaeloff, Elisa Saccon, Ákos Végvári, Rui Benfeitas, Maike Sperk, Marie Ståhlberg, Shuba Krishnan, Kamal Singh, Josef M. Penninger, Ali Mirazimi, Ujjwal Neogi

**Affiliations:** a Public Health Agency of Sweden, Solna, Sweden; b Division of Clinical Microbiology, Department of Laboratory Medicine, Karolinska Institute, Stockholm, Sweden; c Division of Chemistry I, Department of Medical Biochemistry and Biophysics, Karolinska Institutet, Stockholm, Sweden; d National Bioinformatics Infrastructure Sweden (NBIS), Science for Life Laboratory, Department of Biochemistry and Biophysics, Stockholm University Stockholm, Sweden; e Department of Veterinary Pathobiology and the Bond Life Science Center, University of Missouri, Columbia, MO, USA; f Institute of Molecular Biotechnology of the Austrian Academy of Sciences, Vienna, Austria; g Department of Medical Genetics, Life Science Institute, University of British Columbia, Vancouver, Canada; h National Veterinary Institute, Uppsala, Sweden

**Keywords:** SARS-CoV-2, transcriptomics, proteomics, Akt/mTOR/HIF-1, MK-2206

## Abstract

How severe acute respiratory syndrome coronavirus-2 (SARS-CoV-2) infections engage cellular host pathways and innate immunity in infected cells remains largely elusive. We performed an integrative proteo-transcriptomics analysis in SARS-CoV-2 infected Huh7 cells to map the cellular response to the invading virus over time. We identified four pathways, ErbB, HIF-1, mTOR and TNF signaling, among others that were markedly modulated during the course of the SARS-CoV-2 infection *in vitro*. Western blot validation of the downstream effector molecules of these pathways revealed a dose-dependent activation of Akt, mTOR, S6K1 and 4E-BP1 at 24 hours post infection (hpi). However, we found a significant inhibition of HIF-1α through 24hpi and 48hpi of the infection, suggesting a crosstalk between the SARS-CoV-2 and the Akt/mTOR/HIF-1 signaling pathways. Inhibition of the mTOR signaling pathway using Akt inhibitor MK-2206 showed a significant reduction in virus production. Further investigations are required to better understand the molecular sequelae in order to guide potential therapy in the management of severe coronavirus disease 2019 (COVID-19) patients.

## Introduction

The recent emergence of the coronavirus disease 2019 (COVID-19) pandemic caused by severe acute respiratory syndrome coronavirus-2 (SARS-CoV-2) has created a public health emergency across the globe [1–3]. SARS-CoV-2, a single-stranded positive-sense RNA virus, is the seventh coronavirus that infects humans and belongs to the β-coronavirus family. Due to limited knowledge on molecular mechanisms of infection and pathogenesis, there is currently no available vaccine or specific therapeutics to treat or prevent SARS-CoV-2 infection.

Understanding the viral dynamics and host responses to the virus are necessary to design better therapeutic strategies for COVID-19 patients. Within the short period of the pandemic, there are few reports on different levels of omics data (transcriptomics, proteomics, and metabolomics) derived from cell cultures infected with SARS-CoV-2 as well as from patient material that aimed to elucidate potential mechanisms of the host immune response and disease pathogenesis of SARS-CoV-2. However, the steady-state measurements fail to reveal the dynamic changes of the host and viral proteins during the course of the infection. Thus, the temporal changes in gene expression combined with protein synthesis in different phase of the infection have not yet been reported.

To provide a comprehensive assessment of the cellular response to SARS-CoV-2, we performed a time series integrative proteo-transcriptomics analysis in infected Huh7 cells ranging from the early phase of infection until the virus reached its limit of productive infection at ∼72 hours post infection (hpi).

## Materials and methods

***Cells and viruses:*** The SARS-CoV-2 virus was isolated from a nasopharyngeal sample of a patient in Sweden and the isolated virus was confirmed as SARS-CoV-2 by sequencing (Genbank accession number MT093571). The human hepatocyte-derived cellular carcinoma cell line Huh7 (obtained from Marburg Virology Lab, Philipps-Universität Marburg, Marburg, Germany matching the STR reference profile of Huh7 [4]), African Green monkey cell line Vero-E6 (ATCC^®^ CRL-1586^™^) and 16HBE (human bronchial epithelial cell line, obtained from Lena Palmberg, Karolinska Institute) were used.

***Antibodies and drugs:*** Akt (rabbit, Abcam Cat#ab8805), Akt (S473) (rabbit, Abcam Cat#ab81283), mTOR (rabbit, Abcam, Cat#ab32028), mTOR (S2448) (rabbit, Abcam Cat#ab109268), S6K (rabbit, Abcam Cat#ab32529), S6K (T389+T412) (rabbit, Abcam Cat#ab60948), eIF4EBP1(rabbit, Abcam, Cat#Ab32024), eI4EBP1 (T37) (rabbit, Abcam, Cat#ab75767), ENO-1 (rabbit, Abcam, Cat#ab155102) and HIF-1a (Clone 54) (mouse, BD Biosciences, Cat#610959), β-Actin (mouse, Sigma Aldrich, Cat#A5441) SARS-CoV-2 spike S2 (mouse, GeneTex, Cat#GTX632604) and SARS-CoV-2 nucleocapsid (rabbit, Bioserve, Cat#BSV-COV-AB-04). The drugs Wortmannin, MK-2206, Torin-1, BI-D1870, PX-478 and disulfiram was purchased from Selleckchem, US, while SAHA (vorinostat) was purchased from Sigma-Aldrich, US and Rapamycin from Abcam, US.

***Infection and cytotoxicity:*** The infectivity dose of the virus was either determined by plaque-forming assay (for omics studies) or by determining TCID_50_ in Vero-E6 cells. Infection was performed by incubating the cells with virus for one hour at 37°C, 5%CO_2_ in DMEM supplemented with 5% heat-inactivated fetal bovine serum (FBS) followed by removal of virus and replenishing with fresh medium. The virus-mediated cytotoxicity was determined using Viral ToxGlo assay (Promega, US). The virus titer in the supernatant was determined by qPCR targeting either the *E-gene* or *N-gene* using Takara PrimeDirect probe, RT-qPCR mix (Takara Bio Inc, Japan).

***SARS-CoV-2 infection of Huh7 cells for omics:*** Huh7 cells were plated in 6-well plates (2.5 × 10^5^ cells/well) in DMEM (Thermo Fisher Scientific, US) supplemented with 10% heat-inactivated FBS (Thermo Fisher, US). At 90%–95% cell confluence the medium was removed, cells washed carefully with PBS and thereafter either cultured in medium only (uninfected control) or infected with SARS-CoV-2 at a multiplicity of infection (MOI) of 1 added in a total volume of 0.5 mL. After one hour of incubation (37°C, 5%CO_2_) the inoculum was removed, cells washed with PBS and 2 mL DMEM supplemented with 5% heat-inactivated FBS was added to each well. Samples were collected at three different time points, 24, 48 and 72 hours post infection (hpi). Samples were collected for proteomics and RNAseq.

***Total RNA extraction and Quantification of viral RNA****:* The cells (uninfected, 24hpi, 48hpi and 72hpi) were collected by adding Trizol^™^ (Thermo Fisher Scientific, US) directly to the wells. RNA was extracted from SARS-CoV-2 infected and uninfected Huh7 cells and from supernatent using the Direct-zol RNA Miniprep (Zymo Research, US) and quantitative real-time polymerase chain reaction (qRT-PCR) was conducted using TaqMan Fast Virus 1-Step Master Mix (Thermofisher Scientific, US) with primers and probe specific for the SARS-CoV-2 *E gene* following guidelines by the World Health Organization (https://www.who.int/docs/default-source/coronaviruse/wuhan-virus-assayv1991527e5122341d99287a1b17c111902.pdf) as described previously [5].

***Transcriptomics analysis (Illumina RNAseq):*** The samples were sequenced using Illumina NextSeq550 in single-end mode with read length of 75 bases. The raw sequence data were first subjected to quality check using FastQC tool kit version 0.11.8. Illumina adapter sequences and low-quality bases were removed from the raw reads using the tool Trim Galore version 0.6.1. Phred score of 30 was used as cut-off to remove low-quality bases. Quality of the data was again checked after pre-processing to assure high-quality data for further analysis. The pre-processed reads were then aligned against human reference genome version 38 Ensembl release 96. Short read aligner STAR version 2.7.3a was used for the alignment. STAR was executed by setting the parameter soloStrand to Reverse to perform strand specific alignment and rest of the required parameters were set to default. The alignment result was written in sorted by co-ordinate bam format. After the alignment gene level read count data was generated for each sample using the module featureCounts from the software subread version 2.0.0. Read counting was performed by setting attribute type in the annotation to gene_id and strand specificity to reverse. Human reference gene annotation version 38 Ensembl release 96 in gtf format was used for the read counting. Normalization factors were calculated using the R package edgeR [6] from read counts matrix to scale the raw library sizes. Low expression genes with maximum counts per million (CPM) values under 1 per sample were removed from the sample. As recommended in RNAseq, data were transformed to CPM and variance weight was calculated using voom function. Square root of residual standard deviation against log2 CPMs was plotted to verify transformation quality.

***Protein extraction and in-solution digestion:*** The cells (uninfected, 24hpi, 48hpi and 72hpi) were lyzed in lysis buffer (5% glycerol, 10 mM Tris, 150 mM NaCl, 10% SDS and protease inhibitor), NuPAGE^™^ LDS sample buffer (ThermoFisher Scientific,US) was added and the samples were boiled at 99°C for 10 min. Aliquots of cell lysates (150 µL) were transferred to sample tubes and incubated at 37°C for 5 min at 550 rpm on a block heater and sonicated in water bath for 5 min. Each sample was reduced by adding 7 µL of 0.5 M dithiothreitol (DTT) at 37°C for 30 min and alkylated with 14 µL of 0.5 M iodoacetamide for 30 min at room temperature (RT) in the dark. Following the addition of 2 µL of concentrated phosphoric acid and 1211 µL of binding buffer, protein capturing was performed according to the manufacturer’s protocol using S-Trap™ Micro spin columns (Protifi, Huntington, NY). After washing with 150 µL of binding buffer four times the samples were subjected to proteolytic digestion using 1.2 µg trypsin (sequencing grade, Promega) for 2 h at 47°C. Then 40 µL of 50 mM TEAB was added following acidification with 40 µL of 0.2% formic acid (FA) and elution with 40 µL of 50% acetonitrile (AcN)/0.2% FA and the eluents were dried using a Vacufuge vacuum concentrator (Eppendorf, US). The resulted peptides were cleaned up in a HyperSep filter plate with bed volume of 40 µL (Thermo Fisher Scientific, Rockford, IL). Briefly, the plate was washed with 80% AcN/0.1% FA and equilibrated with 0.1% FA. Samples were filtered in the plate and washed with 0.1% FA. Peptides were eluted with 30% AcN/0.1% FA and 80% AcN/0.1% FA and dried in a vacuum concentrator prior to tandem mass tag (TMT) labeling.

***TMT-Pro labeling:*** Dry samples were dissolved in 30 µL of 100 mM triethylammonium-bicarbonate (TEAB), pH 8, and 100 µg of TMT-Pro reagents (Thermo Scientific, US) in 15 µL of dry acetonitrile (AcN) were added. Samples were scrambled and incubated at RT at 550 rpm for two hours. The labeling reaction was stopped by adding 5 µL of 5% hydroxylamine and incubated at RT with 550 rpm for 15 min. Individual samples were combined to one analytical sample and dried ina vacuum concentrator.

***High pH reversed phase LC fractionation and RPLC-MS/MS analysis:*** The TMTPro-labeled tryptic peptides were dissolved in 90 µL of 20 mM ammonium hydroxide and were separated on an XBridge Peptide BEH C18 column (2.1 mm inner diameter × 250 mm, 3.5 μm particle size, 300 Å pore size, Waters, Ireland) previously equilibrated with buffer A (20 mM NH_4_OH) using a linear gradient of 1–23.5% buffer B (20 mM NH_4_OH in AcN, pH 10.0) in 42 minutes, 23.5%–54% B in four minutes and 54%–63% B in two minutes at a flow rate of 200 µL/min. The chromatographic performance was monitored by sampling eluate with a UV detector (Ultimate 3000 UPLC, Thermo Scientific, US) monitoring at 214 nm. Fractions were collected at 30 second intervals into a 96-well plate and combined into twelve samples concatenating eight fractions representing the peak peptide elution. Each combined fraction sample (800 µL) was dried in a vacuum concentrator and the peptides were resuspended in 2% AcN/0.1% FA prior to LC-MS/MS analysis.

Approximately, 2 µg samples were injected in an Ultimate 3000 nano LC on-line coupled to an Orbitrap Fusion Lumos mass spectrometer (MS) (Thermo Scientific, San José, CA). The chromatographic separation of the peptides was achieved using a 50 cm long C18 Easy spray column (Thermo Scientific,US) at 55°C, with the following gradient: 4%–26% of solvent B (2% AcN/0.1% FA) in 120 min, 26%–95% in five minutes, and 95% of solvent B for five minutes at a flow rate of 300 nL/min. The MS acquisition method was comprised of one survey full mass spectrum ranging from *m/z* 350 to 1700, acquired with a resolution of *R* = 120,000 (at *m/z* 200) targeting 4 × 10^5^ ions and 50 ms maximum injection time (max IT), followed by data-dependent HCD fragmentations of precursor ions with a charge state 2+ to 7+ for 2 s, using 60 s dynamic exclusion. The tandem mass spectra were acquired with a resolution of *R* = 50,000, targeting 5 × 10^4^ ions and 86 ms max IT, setting isolation width to *m/z* 1.4 and normalized collision energy to 35% setting first mass at *m/z* 100.

***Peptide identification and preprocessing:*** The raw files were imported to Proteome Discoverer v2.4 (Thermo Scientific) and searched against the *Homo sapiens* SwissProt (2020_01 release with 20,595 entries) and the pre-leased SARS-CoV-2 UniProt (completed with 14 SARS-CoV-2 sequences of COVID-19 UniProtKB release 2020_04_06) protein databases with Mascot v 2.5.1 search engine (MatrixScience Ltd., UK). Parameters were chosen to allow two missed cleavage sites for trypsin while the mass tolerance of precursor and HCD fragment ions was 10 ppm and 0.05 Da, respectively. Carbamidomethylation of cysteine (+57.021 Da) was specified as a fixed modification, whereas TMTPro at peptide N-terminus and lysine, oxidation of methionine (+15.995 Da), deamidation of asparagine and glutamine were defined as variable modifications. For quantification both unique and razor peptides were requested. Protein raw data abundance was first filtered for empty rows with *in house* script and quantile-normalize using R package NormalyzerDE [7]. Principal component analysis (PCA) was applied to explore sample-to- sample relationships. One proteomics samples from the uninfected control were excluded as it turned out to be outlier.

***Statistical analysis:*** Proteomics and transformed transcriptomics data were tested for normality using histograms with normal distribution superimposed. Differential expression through linear model was performed using R package LIMMA [8]. LIMMA supports multifactor designed experiments in microarray, transcriptomics and proteomics. Its features are designed to support small number of arrays. The three infected replicates at 24hpi, 48hpi and 72hpi hours respectively were selected in order to perform an equi-spaced univariate time series analysis. In limma design matrix, separated coefficients were associated with time and replicates in order to extract the difference as a contrast. Moderated paired-t-test using limma with adjustment for replicates was applied. For pairwise comparisons, single factorial design was implemented to fit model with a coefficient for each of our four factors: uninfected, 24hpi, 48hpi and 72hpi. Comparisons were extracted as contrasts. In both analysis, significant differential genes and proteins were selected based on *p* values after Benjamini-Hochberg (BH) adjustment. Genes with alpha value inferior to 0.05 were considered significant.

***Bioinformatics Analysis:*** The transcriptomics and proteomics analysis were performed using all the protein-coding genes and proteins and a gene set of viral processes, response and diseases respectively. The viral response gene set is a catalogue of genes that is known to be involved in viral processes, response and diseases. The catalogue was enriched by mining biological process category of gene-ontology terms, Reactome pathways and gene sets associated with various viral diseases. Gene Ontology terms were selected by keeping, “response to virus (GO:0009615)” as parent term. All child terms of GO:0009615 were selected based on ontology term relationship “is a” and “regulates”. The pathway “Antiviral mechanism by IFN-stimulated genes” and two other events it participates were selected from Reactome database. Gene sets related to 42 virus-associated diseases and six virus-related diseases were selected from “Rare_Diseases_AutoRIF_Gene_Lists” library provided by gene set enrichment tool Enrichr [9]. The viral response gene set contains total of 1517 protein coding genes. After filtering antiviral genes, up and downregulated proteins and transcripts were submitted separately to gene set enrichment analysis (GSEA) using gseapy v0.9.17. R package gplots v3.03 was used to generate heatmaps to display terms associated adjusted *p* values contrasts over conditions.

***Network and community analyses:*** Association analyses were performed by computing pairwise Spearman rank correlations for all features after removing null variant or genes with very low expression (RPKM < 1). Correlations were considered statistically significant at false discovery rate (FDR) < 0.01. Positive correlations were selected and used to build a weighted graph where Spearman ρ was used as weights. All network analyses were performed in igraph [10]. For all networks, diameter, average path lengths, clustering coefficients, and degree distributions were compared with those attained for similarly-sized random networks (Erdős-Rényi models, [11]). Degree centrality was computed for all networks and normalized for network size. Communities were identified by modularity maximization through the Leiden algorithm [12]. Community centrality was computed by averaging node centrality and used to identify the most central communities in each network by degree comparison. Gene set enrichment analysis was performed on each community (*n* > 30) through Enrichr for KEGG Human 2019 where backgrounds were selected based on the node number of each network. Community similarity was computed through hypergeometric testing of overlap between statistically significant KEGG terms for each transcriptomic vs proteomic pair of communities. Throughout, all statistical tests were considered at an FDR < 0.05 unless otherwise stated. All analyses were performed in Python 3.7.

Protein–protein interactions among human proteins were derived from Human Reference Interactome (HuRI). Interactions between human proteins and SARS-CoV-2 viral proteins were obtained from Human Protein Atlas (HPA). Protein interaction network is created using Cytoscape version 3.6.1 [13]. Edge-weighted spring-embedded layout was used for the network. R package gplots 3.03 was used to generate heatmaps to display terms associated *p* values contrasts over conditions. Sankey Plot illustrates most important contribution genes to flow pathways. It was plotted using R package ggalluvial version 0.11.1 [14]. Scatter plots produced using ggplot2 represent the bivariate relationship between proteins and time.

***Western Blot:*** Following 24 hpi and 48hpi with different doses of SARS-CoV-2 infection, the cells were lysed in 2x NuPage LDS sample buffer (Thermo Scientific, US) followed by boiling at 95°C for ten minutes to inactivate the virus. The protein concentration was evaluated by Pierce^™^ 660nm Protein Assay kit (Thermo Scientific, US). Evaluation of protein expression was performed by running 20 μg of total protein lysate on NuPage Bis Tris 4%–12%, gels or NuPage Tris-Acetate 3%–8% gels (Invitrogen, Carlsbad, CA, USA). Proteins were transferred using iBlot dry transfer system (Invitrogen, Carlsbad, CA, USA) and blocked for one hour using 5% milk or bovine serum albumin (BSA) in 0.1% PBSt (0.1% Tween-20). Subsequent antibody incubation was performed at 4°C overnight or for one hour at room temperature for β-Actin. Membranes were washed using 0.1% PBSt and secondary antibody was incubated for one hour at room temperature using Dako Polyconal Goat Anti-Rabbit or Anti-Mouse Immunoglobulins/HRP (Aglient Technologies, Santa Clara, CA, USA). Membranes were washed using 0.1% PBSt and proteins were detected using ECL or ECL Select (GE Healthcare, Chicago, IL, USA) on ChemiDoc XRS+ System (Bio-Rad Laboratories, Hercules, CA, USA). The western blot analysis was performed by using antibodies targeting Akt, p-Akt, mTOR, p-mTOR, S6K, p-S6K, 4E-BP1, p-4E-BP1and HIF-1α. Viral RNA was quantifed from cell supernatent in all the time points as a confirmation of the infection by using Takara PrimeDirect probe, RT-qPCR mix (Takara Bio. Inc, Japan).

***Drug treatment and virus infectivity:*** Inhibitors and modulators of PI3K and mTOR signaling pathways, namely Wortmannin, MK-2206, Torin-1, Rapamycin, BI-D1870, PX-478, Disulfiram and SAHA (vorinostat) were reconstituted in DMSO and cytotoxicity at different concentrations and time points (24h and 48 h) was determined in Huh7 cells using alamarBlue (Invitrogen, US). Changes in expression of different components of the pathway at effective and non-toxic concentration for 24 h and 48 h are shown in supplementary Figure S1. To determine the effect of these drugs on virus replication, Huh7 cells were pretreated with the drugs for twelve hours at a concentration that was suitable for 48 h incubation in DMEM supplemented with 2%FBS. The cells were infected with SARS-CoV-2 at MOI of 0.1 in presence of the drugs for one hour. The virus was removed, and the cells were further incubated for 24 h in presence of the drugs in DMEM supplemented with 2% FBS. DMSO was used as a control. The virus infectivity was determined by qPCR in both the supernatant and in the cells.

***RNAScope^®^ assay:*** RNAScope^®^ assay was performed targeting HIF-1α using the probe RNAscope^®^ Probe-Hs-HIF1A-C2 (ACD Bio, US) (Cat# 605221-C2) as described by us previously [15].

***Data and Code Availability.*** The raw RNAseq data can be obtained from the SRA using the project id. PRJNA627100. Proteomics data can be obtained from https://zenodo.org/record/3754719#.XqgnSy2B3OQ. All the codes are available at github: https://github.com/neogilab/COVID19

## Results

**Figure 1.**
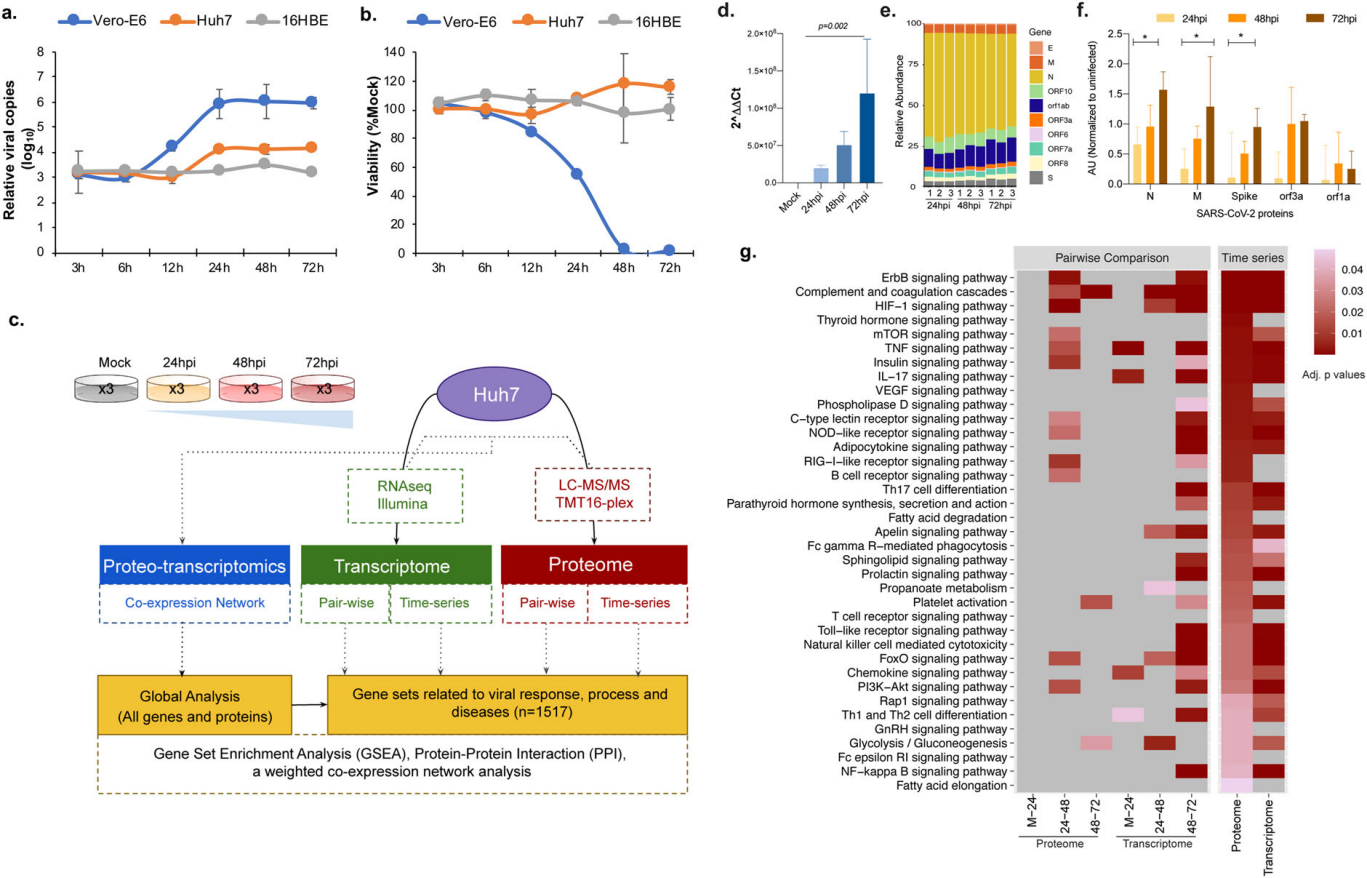
Infection dynamics and proteo-transcriptomics in Huh7 cell line. (a) Vero-E6, Huh7 and 16HBE cell lines were challenged with SARS-CoV-2 at 1 MOI. Viral supernatant samples were harvested at 3 h post infection (hpi), 6hpi, 12hpi, 24 hpi, 48hpi and 72hpi. Viral load was determined by quantitative RT-PCR targeting the N gene of SARS-CoV-2. The viral load at each time point was compared with baseline viral load at 3hpi. (b) The cell viability at each time point was measured by viralToxGlo assay. The viability at each time was determined in comparison to the uninfected control. (c) Brief methodology of the omics experiments. (d) Viral RNA quantification in the Huh7 infected cells using qPCR targeting the E gene of SARS-CoV-2. (e) Detected viral genes and open reading frame in the RNAseq experiment. (f) Temporal dynamics of detected proteins in the Huh7 cells by tandem mass tag-labelled mass spectrometry (TMT-MS). (g) Gene set enrichment analysis using the genes related to viral response, process and diseases in single omics level by pairwise comparative analysis and time series analysis at individual omics level. Significant (adjusted *p* values) KEGG terms enriched for upregulated genes are represented as heatmap. The lower adjusted *p* values are shown in dark red color and higher ones with light red color, non-significant pathways are represented in grey color.

***Dynamics of the SARS-CoV-2 infection in in different cell lines:*** Vero-E6 (ATCC^®^ CRL-1586^™^), 16HBE and Huh7 cells were infected with SARS-CoV-2 at MOI 1.0 [5]. Cells were collected at 3hpi, 6hpi, 12hpi, 24hpi, 48hpi, and 72hpi and subjected to viral RNA quantification by quantitative PCR and to cytotoxicity assay. The viral copy number dynamics indicated that Vero-E6 showed an increase in viral copy numbers at 12hpi, while for Huh7 the viral load showed an increase at 24hpi (Figure 1(a)). No apparent productive replication was observed in 16HBE cells. The cytotoxicity was observed only in Vero-E6 (Figure 1(b)). Based on these findings we used Huh7 infected with SARS-CoV-2 and collected infected cells 24hpi, 48hpi, and 72hpi for temporal transcriptomics by Illumina NextSeq550 and proteomics by tandem tag labelled mass spectrometry (TMT-MS). Transcriptomics, proteomics and proteo-transcriptomics data were further analysed using in-depth bioinformatics (Figure 1(c)). qPCR targeting the envelope (E) gene of the SARS-CoV-2 identified a gradual increase in cellular viral RNA over time (*p*<0.05, repeated measure ANOVA) (Figure 1(d)). RNAseq analysis detected viral RNA at all the time points; 24hpi, 48hpi and 72hpi (Figure 1(e)). The TMT-based quantitative proteomics also identified statistically significant increase (*p*<0.05, repeated measure ANOVA) in SARS-CoV-2 proteins nucleocapsid (N), membrane (M) and spike (S) over time (Figure 1(f)). Thus, SARS-CoV-2 exposure of Huh7 cells resulted in effective infection that over time lead to enhanced viral RNA and viral protein production, which is required to assemble viral progeny.

***Host cellular response against SARS-CoV-2 in vitro***. After having established effective SARS-CoV-2 infections, we assessed the cellular host response to the virus infection. We found that 2622 genes and 1819 proteins were increased whereas 2856 genes and 1743 proteins were decreased significantly (false discovery rate <0.05) over the time despite distinct coverage (19997 protein-coding genes vs 7757 proteins quantified) compared to control. We next performed gene set enrichment analyses using the differentially expressed genes/proteins that are related to viral response, process and diseases (targeted analysis) obtained from Gene Ontology (GO), REACTOME and “Rare_Diseases_AutoRIF_Gene_Lists” library and mapped to KEGG (Human_2019) terms. Figure 1(g) shows a heatmap of significantly enriched KEGG terms that are dysregulated in both our proteomics and transcriptomics analysis using pairwise and time series analysis in uninfected and SARS-CoV-2 infected Huh7 cells. Of note, the downregulated genes did not identify any KEGG term with adjusted *p* value <0.05. Among the most significantly upregulated pathways, mining both proteomics and transcriptomics data, were pathways associated with cell proliferation and apoptosis, such as ErbB, PI3K-Akt, HIF-1, and mTOR signaling, and pathways that are related to innate immune responses such as TNF, NOD-like receptor (NLR) and RIG-I signaling (Figure 1(g)). The majority of the changes were observed between 24hpi and 48hpi. In addition, we observed an upregulation in platelet activation, complement cascades, FOXO signaling, and glycolysis (Figure 1(g)), indicating that the SARS-CoV-2 infection induces pathways linked to thrombosis and metabolism.

**Figure 2.**
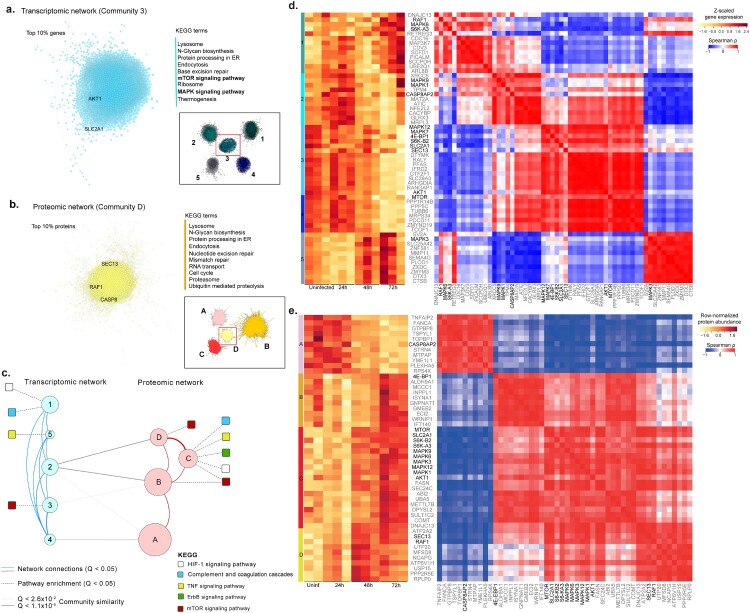
Network analysis using genes and proteins. Analysis of the most central communities in each network highlights key KEGG terms (right) among the top 10% associated genes and proteins. The top 10% correlations (Spearman rho > 0.95, FDR < 0.05) were selected in the most central community in transcriptomic (a) and proteomic (b) networks (inset) based on mean normalized degree. The top KEGG terms associated with each of the two communities (FDR < 0.05) are highlighted, as well as genes that had been previously found in Figure 1(g). (c) A proteo-transcriptomic network analysis highlights coordinated expression and functional changes in response to viral infection. Communities (circles) in transcriptomic and proteomic networks, where node size is proportion to the number of elements (728 - 2519). Edges indicate association (Q<0.05) with KEGG terms (dashed), network edges (solid red and blue), or community similarity (solid gray). (d) Gene expression and co-expression among key genes and top correlated and central genes in each community identified based on a transcriptomic network (communities 1-5). (e) Protein abundance (A) and correlations (B – C) among key proteins and top correlated and central proteins in each community identified based on a proteomic network (communities A-D). For each community we identified selected the top ten genes (grey labels), ranked by their median centrality (median ranked degree, betweenness, closeness and eccentricity centralities), among the top 10% correlated gene in each community. Key proteins, previously associated with HIF-1α, mTOR, MAPK signaling and other top pathways, are highlighted in black (Figure 1(g)). Spearman rank correlations were computed for all genes and excluded if not statistically significant (Figure S2, FDR < 0.01).

***Proteo-transcriptomics co-expression network:*** To further capture the patterns of expression changes in response to the SARS-CoV-2 infection, we performed a weighted co-expression network analysis on both transcriptomic and proteomic datasets using all genes and proteins detected, their functional assignments, and the top genes (Figure 2(a)) and proteins (Figure 2(b)) in key network elements. For transcriptomic and proteomic networks (adjusted *p* value <0.01, Spearman ρ > 0.83), we identified a set of five transcriptomics and four proteomics communities of strongly interconnected genes and proteins (Figure 2(c)). These communities were also validated against random networks. Characterization of these communities again highlighted several pathways of interest including HIF-1, mTOR, and TNF signaling previously observed in our pairwise comparisons and time series analyses (Figure 1(g)). Ranking of all communities based on their centrality further identified those that display a higher number of central genes/proteins, i.e. communities that exhibit a larger number of associated genes/proteins and thus capture most coordinated expression changes and hence are predicted to robustly influence network behavior. The two most central communities (Figure 2(a,b)) entail several genes associated with AKT1, SLC2A1 (part of HIF-1 signaling), RAF1 (part of MAPK signaling), SEC13 (part of mTOR signaling) and Caspase 8 (CASP8; part of TNF signaling). Functional enrichment analysis indicated that these two communities were associated (adjusted *p*< 0.05) with mTOR and MAPK signaling, Lysosomal and Proteasome-related processes, and cell cycle control. Importantly, we found that MAPK, AKT1, and mTOR showed cumulative expression changes through time (Figure 2(d,e), Figure S2); moreover, they were co-expressed (adjusted *p*<0.05) with several other genes/proteins, including the most central ones in each community (Figure 2(d,e), Figure S2), further highlighting the importance of these genes/proteins in coordinating the global response to infection. Finally, we found significant (adjusted *p*<0.05) functional overlap between three transcriptomic and three proteomic communities (communities 2, 3, 4; and communities A, B, D; Figure 2(c)), thus pointing to common biological responses at the proteo-transcriptomic levels. These intersections included mTOR signaling (community 3 vs B), oxidative phosphorylation (communities 2,4 vs B) and thermogenesis (community 2,3,4 vs B), which are simultaneously found among similar communities in transcriptomic and proteomic networks. Our functional and network community analyses identified common host cell genes (AKT1, MAPK) and biological pathways (mTOR, MAPK and HIF-1 signaling) that were upregulated in SARS-CoV-2 infected Huh7 cells.

**Figure 3.**
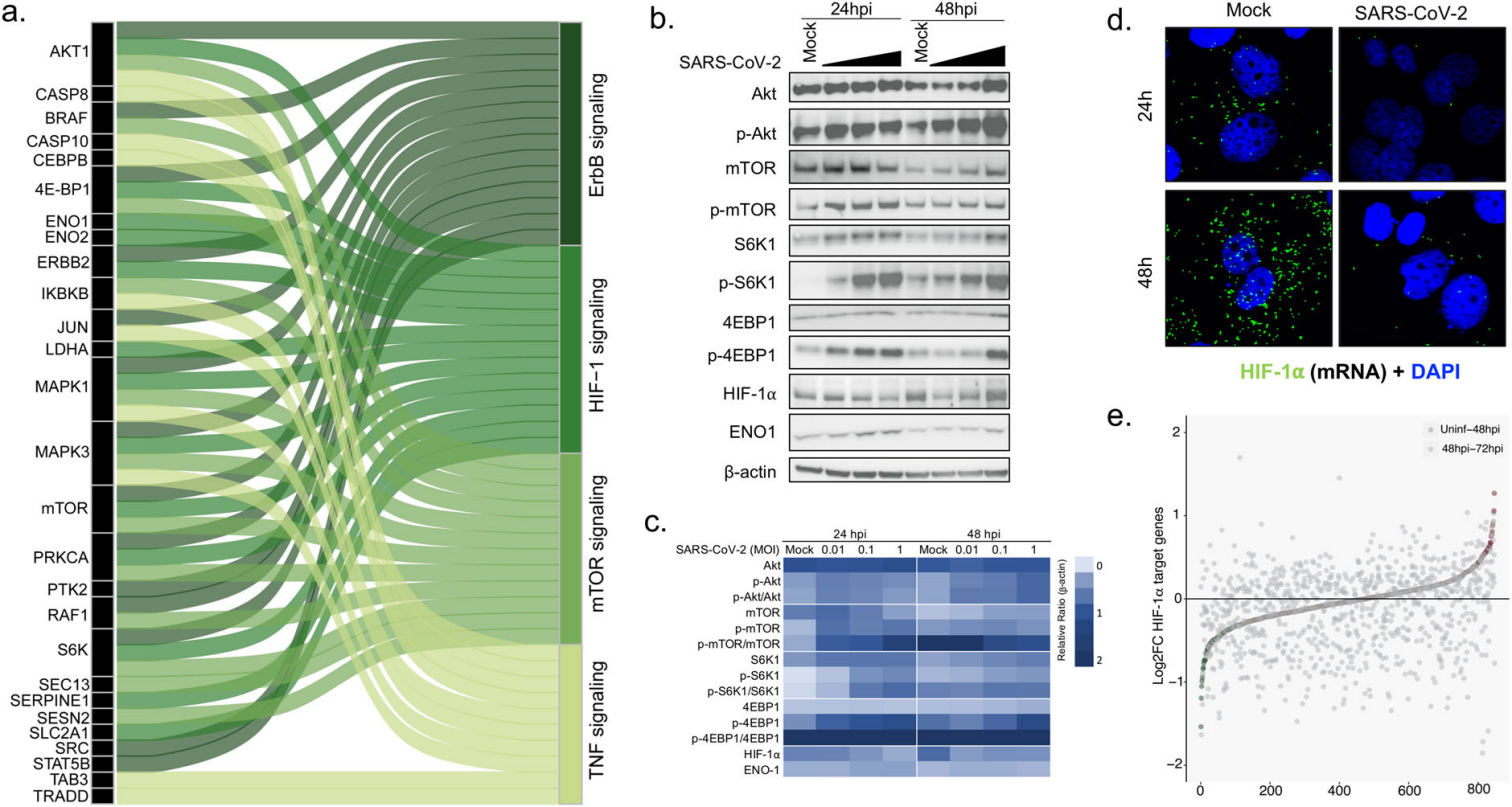
SARS-CoV-2 modulates Akt-mTOR-HIF signaling. (a) Top four pathways, ErbB signaling, HIF-1 signaling, mTOR signaling and TNF signaling were selected and, together with proteins that are altered in the infection course, represented as Sankey Plot in order to illustrate the most important contribution to the flow of each pathway. (b) Huh7 cells were infected with SARS-CoV-2 at MOI of 0.01, 0.1 and 1 and cells were harvested at 24hpi and 48hpi. The representative western blots with indicated antibodies are shown. (c) A heatmap with the densitometric protein quantification is shown. All the MOI’s at each timepoint were normalized to β-actin. (d) HIF-1α m-RNA transcripts were visualized using RNAscope in mock infected and SARS-CoV-2 infected (MOI 1) Huh7 cells at 24hpi and 48hpi. (d) Graph showing upregulation and downregulation of HIF-1α target genes at 48hpi to 72hpi in the transcriptomics data as a measure of fold change. Significantly upregulated and downregulated genes are marked in red and green respectively.

***Dysregulated proteins and effector molecules in Akt/mTOR/HIF-1 signaling****.* The top four identified pathways, ErbB, PI3K-Akt, HIF-1, and mTOR signaling showed overlap of several proteins like AKT1, mTOR, MAPK, 4E-BP1, and S6K as represented in Sankey plot (Figure 3(a)). Since all the top identified pathways converge at mTOR signaling, we wanted to investigate whether SARS-CoV-2 infections indeed change expression of upstream and critical effector molecules of the mTOR/HIF-1 signaling pathway. The mTOR pathway is involved in various biological functions and several viruses hijack this pathway to promote their own replication in different ways [16]. Given that the major changes were observed between 24hpi and 48hpi and the mTOR pathway is modulated by the nutrition, we restricted our analysis to up to 48hpi. To this end the expression of different components of the Akt-mTOR-HIF-1 signaling was assessed during SARS-CoV-2 infection in a dose-dependent manner using MOI of 0.01, 0.1 and 1. The infection dynamics measured by SARS-CoV-2 RNA in the cell culture supernatant is shown in supplementary Figure S3. The western blot results supported the omics findings and showed an activation of the Akt-mTOR pathway as observed by a dose-dependent increase in phosphorylation of Akt, mTOR, 4E-BP1 and S6K1 (Figure 3(b,c)). The activation was more prominent at 24hpi suggesting that SARS-CoV-2 activates Akt-mTOR signaling during the initial phase of infection. However, it was surprising to note that in spite of activation of the effectors of mTOR pathway, HIF-1α protein levels were rapidly reduced following SARS-CoV-2 infections (Figure 3(b,c)). Furthermore, using RNAscope we observed that SARS-CoV-2 infected cells showed significantly lower level of HIF-1α mRNA both at 24hpi and 48hpi as compared to the mock infected cells (Figure 3(d), Figure S4). We also checked the overlapping differently abundant proteins between the Huh7 cells (uninfected vs 48hpi) and Caco-2 (uninfected vs 24hpi) reported by Bojkova *et al.* [17] and observed 602 proteins overlapping between the two cell lines of which several are part of mTOR, PI3K-Akt and HIF-1 signaling pathways (Figure S5).

To determine the effect of HIF-1α suppression in SARS-CoV-2 infected we studied the effect of SARS-CoV-2 infection on target genes of HIF-1α (n=1256, as reported [18]) in our transcriptomics data. To observe the early effect, we compared uninfected with 48hpi and observed 61 (5%) genes differentially expressed (Figure 3(e) and supplementary data 1). However, while comparing the differential expressed genes between 48hpi and 72hpi, we observed that 306 (24%) genes were significantly differentially expressed of which 68% (208/306) were also downregulated (adjusted *p* values <0.05) (supplementary data 1).This could be an effect of HIF-1α downregulation.

**Figure 4.**
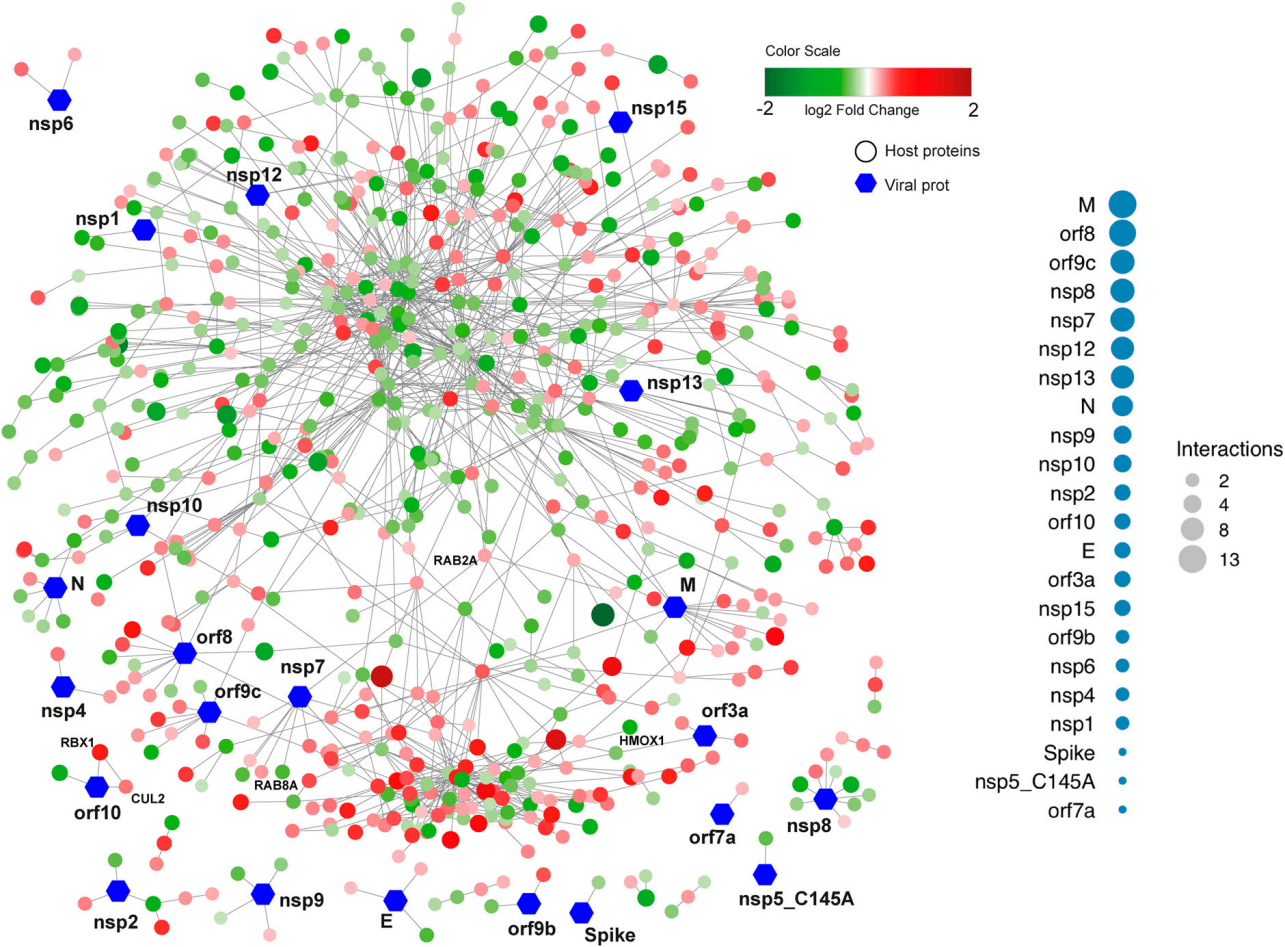
Network visualizing protein interactions among significantly changing proteins between samples at 24hpi and 48hpi, and SARS-CoV-2 viral proteins. Green color nodes represent decreased proteins at 48hpi and red colored proteins represent increased proteins at 48hpi. Size of the nodes are relative to their log2 fold change. Hexagonal shaped nodes denote SARS-CoV-2 viral proteins. The edges are derived from Human Reference Interactom (HuRI) and SARS-CoV-2 entry in Human Protein Atlas.

***Drug repurposing and viral host protein interactions of the dysregulated proteins.*** To repurpose antiviral drugs targeting host-viral interactions is an attractive strategy to find drugs that might work against COVID-19. Therefore, we assessed protein–protein interactions including both host protein and SARS-CoV-2 protein associations obtained from the Human Protein Atlas (https://www.proteinatlas.org/humanproteome/sars-CoV-2) [19]. Proteins that were significantly increased between 24h and 48h after SARS-CoV-2 infection (Figure 4) were arbitrarily assigned to early responses. A total of 108 host-viral protein interactions were observed. The majority of the interactions was observed with the viral protein M (13 interactions), followed by orf8 (12 interactions), orf9c (11 interactions), nsp7, nsp8 (9 interactions) and nsp12 (8 interactions). Interestingly, while mapping these proteins with the pathway we observed that three HIF-1 pathway-associated proteins were markedly altered in the infected cells: Heme Oxygenase 1 (HMOX1), which interacts with orf3a, decreased over time, whereas Cullin 2 (CUL2) and Ring-Box 1 (RBX1), both of which interact with orf10, increased over time. In addition, we found that the two Ras-associated proteins RAB8A and RAB2A interact with nsp7. Furthermore, we found that Receptor-interacting serine/threonine-protein kinase 1 (*RIPK1*), involved in NF-κB, NLR, RIG-I-like receptor and TNF signaling [20], interacted with nsp12 and was significantly enriched over time (Figure 4).

**Figure 5.**
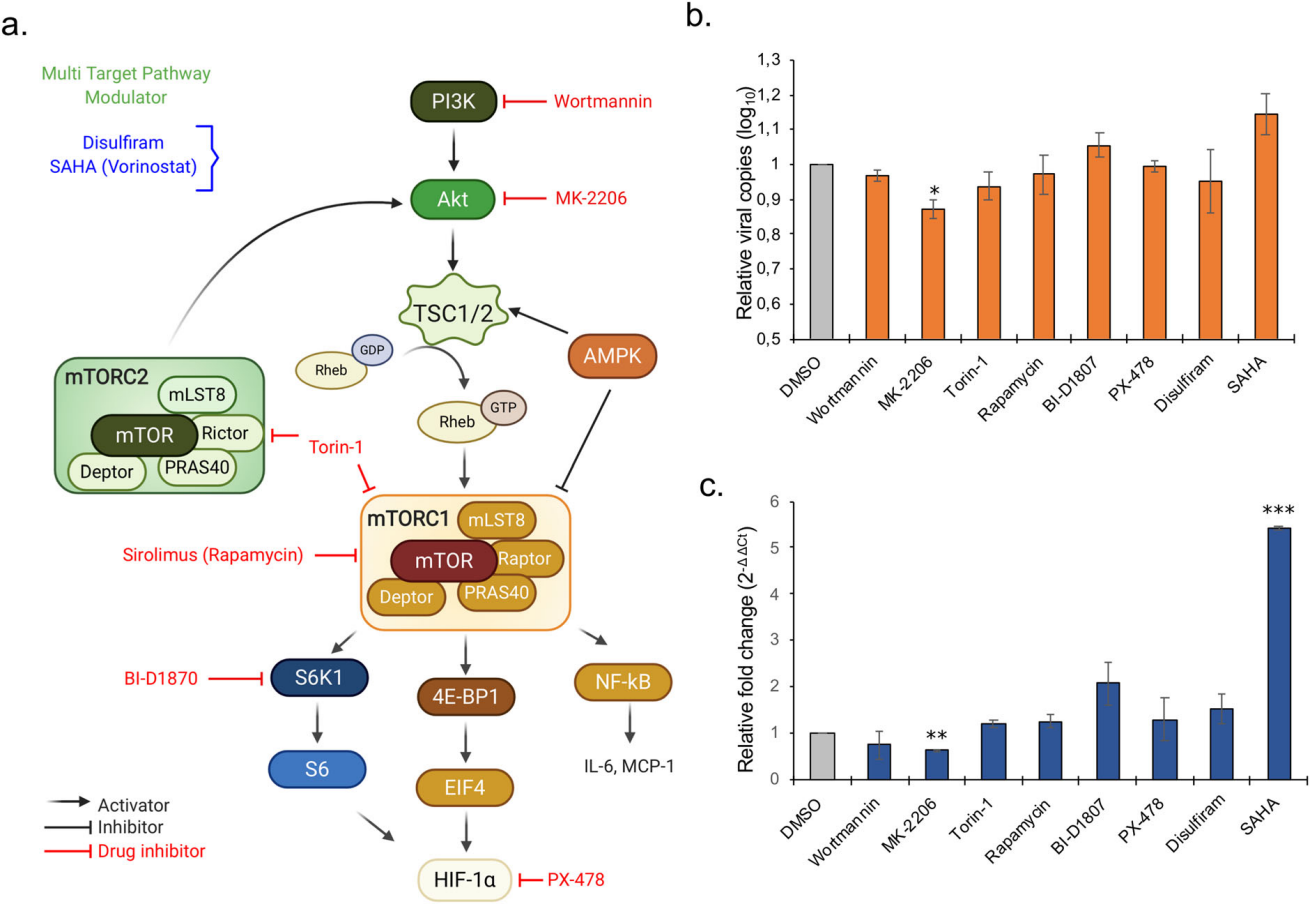
Repurposing of approved drugs targeting Akt/mTOR/HIF-1 signaling pathway. (a) Schematic representation of Akt-mTOR-HIF-1 signaling. Only key proteins of the pathway and the inhibitors (red) and modulators (blue) used in the study are shown. (b-c) Antiviral assay for the inhibitors and modulators was performed in duplicates. (b) The bar graph shows relative viral copies compared to mock infection in the supernatant after 24 hpi. (c) The bar graph shows relative fold change of viral RNA based on the Ct-values in the cellular RNA after 24 hpi.

***Modulation of Akt/mTOR/HIF-1 pathway.*** Our data indicate a role of Akt/mTOR/HIF-1 in the cellular response to the SARS-CoV-2 infection, suggesting that drugs blocking this pathway could possibly be repurposed for COVID-19 patients. We have used eight drugs that either inhibit PI3K (Wortmannin; 12.5 μM), Akt (MK-2206; 3.12 μM), mTORC1 (Rapamycin; 6.25 μM), mTORC1/2 (Torin-1; 1.562 μM), S6K1 (BI-D1870; 3.12 μM) and HIF-1a (PX-478; 12.5 μM) or that modulate the PI3K/Akt/mTOR/HIF pathway using disulfiram (3.12 μM) and HDAC inhibitor (SAHA/vorinostat; 1.56 μM) [21]. (Figure 5(a)). All the drugs showed a differential expression of the proteins associated with Akt/mTOR/HIF signaling. Our data indicate that inhibition of Akt by MK-2206 can significantly suppress SARS-CoV-2 infection (*p*<0.05) and replication as evident by decreased viral transcripts in cells and in supernatant (Figure 5(b,c)). While no other direct inhibitor significantly reduced the infection, we observed a marginal increase in infection by blocking S6K1 with BI-D1870. HDAC inhibitor that can modulate the pathway at various levels significantly increased SARS-CoV-2 infection (Figure 5(b,c)). This led us to speculate that SARS-CoV-2 can modulate the Akt/mTOR/HIF signaling at various levels to promote its infection.

## Discussion

In this study using the integrated proteo-transcriptomics approach we identified four pathways, ErbB, HIF-1, mTOR and TNF signaling, among others that were markedly modulated during the course of the SARS-CoV-2 infection *in vitro*. Western blot validation of the upstream and downstream effector molecules of mTOR revealed activation of the Akt/mTOR signaling and suppression of HIF-1α following SARS-CoV-2 infection. The data therefore points towards dysregulation of Akt/mTOR/HIF-1 signaling cascades, which could be a potential target for COVID-19 therapeutic interventions.

The mTOR signaling pathway is known to regulate apoptosis, cell survival, and host transcription and translation and it can be hijacked by several RNA viruses like influenza virus and coronaviruses other than SARS-CoV-2 [22–25]. PI3K activation results in Akt phosphorylation and subsequent activation of mTOR. Through a cascade of events, mTORC1 and Akt activate 4E-BP1 and eIF4 complex followed by translation of effector protein HIF-1α that initiates host transcription and translation of specific genes. Another pathway that changed over time was the TNF signaling pathway. TNF signaling is also interlinked with HIF-1 signaling and can induce HIF-1α through Akt and MAPK activation [26]. Of note, specific proteins dysregulated in the TNF signaling pathway were caspase 8, caspase 10 [27], and CCAAT/enhancer-binding protein beta (CEBPB) [28], which are linked to interferon (IFN) signaling and NF-κB signaling pathways. Previous studies on coronaviruses suggest a critical role of the IFN response, in particular IFN-β [29,30]. This is also reflected in our findings since SARS-CoV-2 infection resulted in significantly dysregulated RIG-I, NLR and NF-κB pathways. These needs, however, further evaluation. All these pathways have been linked to the IFN response.

Viruses can target various components of mTOR signaling to promote their replication. The activation of Akt/mTOR signaling during SARS-CoV-2 infection could be to sustain protein synthesis by increased accession to translation components and by overcoming infection-associated stress by blocking autophagy and apoptosis [31]. Thus, similar to other viruses hijacking the Akt/mTOR pathway such as the highly pathogenic 1918 influenza virus [22] and the Middle East respiratory syndrome coronavirus (MERS-CoV) [25], dysregulation of the mTOR pathway might enable SARS-CoV-2 to enhance its pathogenicity. Although SARS-CoV-2 infection promoted activation of the Akt/mTOR pathway we observed a suppression of HIF-1α both at the protein and the transcript level. It has been shown that absence of HIF-1α can promote replication of influenza A virus and severe inflammation mediated via promotion of autophagy [32].

There are a few other studies where MS-based proteomics were performed in SARS-CoV-2 infected Caco-2 cells (human epithelial colorectal adenocarcinoma cells) [17] and Vero-E6 cells [33]. In the study by Bojkova *et al.* SARS-CoV-2 was found to be rapidly replicating in Caco-2 cells showing a prominent cytopathic effect by 24 h. While they observe SARS-CoV-2 to increase host-translation machinery, spliceosome and metabolic pathways, they also find HIF-1 signaling to be one of the top pathways that is modulated during the infection [17]. This overlaps with our finding in Huh7 cell line. However, the study by Zecha *et. al* [33] in Vero-E6 infection model showed only partial overlap between the significantly regulated proteins in the Vero-E6 and Caco-2 cell lines. However, it must be noted that, since Vero-E6 is a monkey cell line, it may not be a physiologically relevant cell line for SARS-CoV-2 infection. Furthermore, other than differences in the cell lines in these studies, there could be experimental differences with respect to the methodology, infecting dose and virus strain. The differences are evident with discrepant results observed with respect to infectivity in different studies. While the study by Bojkova *et al.* [17] shows rapid infectivity in Caco-2, the study by Zecha *et. al* [33] and another study by Chu *et al.* [34] shows poor infectivity in Caco-2 even with higher MOI and the later showing no apparent cytopathic effect.

Based on the proteomics data several studies have suggested repurposing of drugs targeting cellular pathways that are affected by SARS-CoV-2. A recent drug target network analysis based on potential human coronavirus and host interactions predicted that sirolimus (also known as rapamycin), which targets mTOR, could be repurposed [35]. Sirolimus was shown to inhibit MERS-CoV infection by 60% in mice [25]. Some studies have shown that everolimus, another mTOR inhibitor, and sirolimus are weakly active against influenza A virus [36,37]. Everolimus delayed death but was not able to reduce mortality in lethal mouse infection model of influenza A (H1N1 and H5N1) [36]. Sirolimus has been reported to block viral protein expression and virion release, improving the prognosis in patients with severe H1N1 pneumonia and acute respiratory failure [38]. On the other hand it was shown to negatively affect the lung pathology probably due to its immunosuppressive effect [37]. Furthermore, rapamycin treatment was also shown to degrade antiviral barriers and could thus be potentially harmful in pathogenic viral infections [39]. In our infection model mTOR inhibitors rapamycin and Torin-1 failed to block viral infection. However, Akt inhibitor MK-2206 showed significant inhibition of viral replication. This is also supported by a recent study by Gassen et. al. where an inhibitory effect of MK-2206 on SARS-CoV-2 was observed in VeroFM cells [40]. We have observed that inhibition of Akt by MK-2206 causes stabilization of mTORC1 (Figure S1), and this may be due to reduced inhibition of mTORC1 by TSC-2. At the same time, SAHA showed inhibition of mTORC1 (Figure S3) and caused increased replication of SARS-CoV-2 suggesting a role of mTOR in virus regulation. Gassen *et. al* has suggested that inhibitory effect of MK-2206 could be a result of enhanced autophagy due to stabilization of Beclin-1 [40]. It is also important to note that along with the mTOR inhibitors, the upstream PI3K inhibitor Wortmannin and downstream S6K1 inhibitor BI-D1870 did not show any significant inhibition of the virus, rather BI-D1870 seemed to promote virus replication. Therefore, our data is suggestive of a dynamic interplay between the virus and the mTOR signaling. It is likely that SARS-CoV-2 can regulate the pathway at various levels and the modulation of these pathways could very well be dependent on viral load and duration of infection. MK-2206 was also shown to effectively inhibit the pH1N1 influenza virus by inhibiting endocytic uptake of the virus due to inhibition of Akt [41]. MK-2206 does not directly act on the virus and targets complex cellular pathways that are modulated by the virus, thus the precise mechanism needs to be carefully interrogated.

In COVID-19 patients the severity of the disease is associated with a cytokine storm with markedly increased expression of interleukin 6 (IL-6) in the serum of severe cases [42]. Interestingly, IL-6 can activate mTOR in a STAT3 dependent or independent manner [43]. Therefore, mTOR inhibitors could also act as a key immune regulator of the “cytokine storm” through the mTOR-NLRP3-IL-1β axis of the IL-6 pathway. Whether the currently proposed drugs targeting the Akt/mTOR pathway can be indeed repurposed for COVID-19 therapies now needs to be tested carefully in *in vitro* SARS-CoV-2 infection models and in *in vivo* COVID-19 disease models as improper regulation may pose a detrimental effect. The ongoing phase I (SirCo-1, NCT04371640), phase II (The SCOPE trials, NCT04341675) and phase III (NCT04409327) trials that target mTOR in the severe COVID-19 patients will provide more information.

There are some limitations of our study. First, we only used the Huh7 cell line but SARS-CoV-2 has been reported to be cultured in Vero-E6, Vero CCL81, Caco2, Calu-3 or HEK-293T cells with contradictory reports of cytopathic effect. We failed to observe any cytopathogenicity in 16HBE as well as in other cell lines like HEK-293T, H441 and HULEC (data not shown). While SARS-CoV-2 exerts rapid cytopathic effects in Vero-E6 cells (within 24hpi), viral replication is probably slower in Huh7 cells and it did not promote cytopathogenicity in our model, allowing to study host-cellular responses for up to three days after viral challenge [44]. Moreover, an earlier study used Huh7 cells to identify the transcriptomics signature of early cellular responses to SARS-CoV and HCoV-229E infections [45]. However, the observed effects could be cell type-specific or strain-specific. Moreover, SARS-CoV-2 has a propensity to mutate and our experiments were performed with only one virus strain isolated from a Swedish patient. Of note, our virus isolate has close sequence similarity to the initial strains circulating in Wuhan, China.

In conclusion, we observed marked alterations of Akt/mTOR/HIF-1 signaling at the proteo-transcriptomic levels in Huh7 cells in response to SARS-CoV-2 infection, though the exact mechanistic role of these changes remains to be elucidated. Targeting Akt-mTOR signaling could be an attractive candidate for a potential therapy, alone or preferably combined with antivirals, for the management of COVID-19 patients and needs further evaluation.

## Supplementary Material

Supplemental Material

Supplimentary_Data_1.xlsx
