# Supplementary material for: Dysregulation in Akt/mTOR/HIF-1 signaling identified by proteo-transcriptomics of SARS-CoV-2 infected cells
Source: Emerg Microbes Infect. 2020 Jul 31;9(1):1748–60. doi: 10.1080/22221751.2020.1799723 (PMC7473213; doi:10.1080/22221751.2020.1799723)
Supplement: Supplemental Material [file TEMI_A_1799723_SM2364.pdf]

Supplemental Information

Dysregulation in Akt/mTOR/HIF-1 signaling identified by proteo-transcriptomics of SARS-CoV-2 infected cells

Sofia Appelberg[1#], Soham Gupta[2#], Sara Svensson Akusjärvi[2], Anoop T Ambikan[2], Flora Mikaeloff[2], Elisa Saccon[2], Ákos Végvári[3], Rui Benfeitas[4], Maike Sperk[2], Marie Ståhlberg[3], Shuba Krishnan[2], Kamal Singh[2,5], Josef M. Penninger[6,7], Ali Mirazimi[1,2,8*], Ujjwal Neogi[2,5,*]

[1]*Public Health Agency of Sweden, Solna, Sweden*

[2]*Division of Clinical Microbiology, Department of Laboratory Medicine, Karolinska Institute, ANA Futura, Campus Flemingsberg, Stockholm, Sweden.*

[3]*Division of Chemistry I, Department of Medical Biochemistry and Biophysics, Karolinska Institutet, Stockholm, Sweden*

[4]*National Bioinformatics Infrastructure Sweden (NBIS), Science for Life Laboratory, Department of Biochemistry and Biophysics, Stockholm University, S-10691 Stockholm, Sweden*

[5]*Department of Veterinary Pathobiology and the Bond Life Science Center, University of Missouri, Columbia, MO 65211, USA*

[6]*Institute of Molecular Biotechnology of the Austrian Academy of Sciences, Dr. Bohr-Gasse 3, 1030 Vienna, Austria.*

[7]*Department of Medical Genetics, Life Science Institute, University of British Columbia, Vancouver, V6T 1Z3, British Columbia, Canada.*

[8]*National Veterinary Institute, Uppsala, Sweden*

[#]*Equal contribution*

*Corresponding Authors: Ali Mirazimi (ali.mirazimi@folkhalsomyndigheten.se), Ujjwal Neogi (ujjwal.neogi@ki.se)

a.

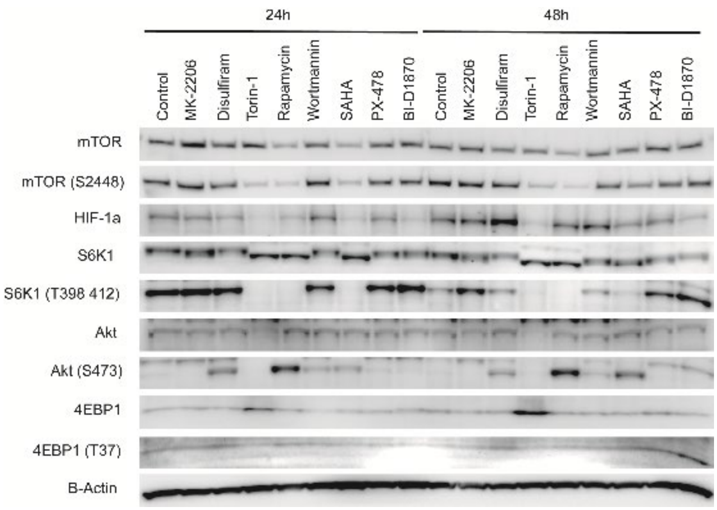

b.

Drugs	24h Conc. (μM)	48h Conc. (μM)
MK-2206	6.25	3.125
Disulfiram	6.25	3.125
Torin-1	3.125	1.5625
Rapamycin	12.5	6.25
Wortmannin	12.5	12.5
SAHA	3.125	1.5625
PX-478	12.5	12.5
BI-D1870	6.25	3.125

Figure S1 – (a) Expression of different components Akt-mTOR-HIF signaling pathway in presence of different inhibitors and modulators at 24h and 48h of treatment in Hu7 cells determined by western blot with indicated antibodies. (b) The concentration of the drugs at each time point are shown.

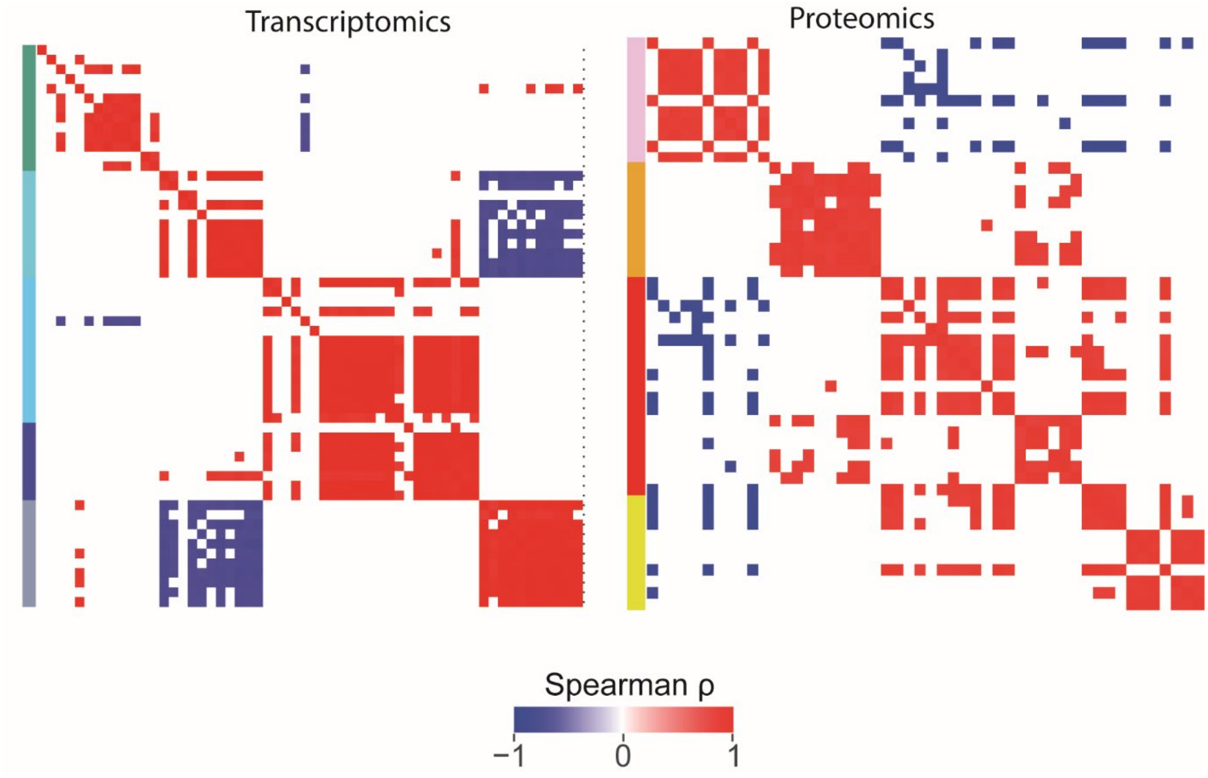

Figure S2 – Gene co-expression and protein correlation analysis among key genes and top correlated and central genes in each community identified based on a transcriptomic network (communities 1-5) and central proteins in each community identified based on a proteomic network (communities A-D). Spearman rank correlations for statistically significant (FDR < 0.01) were presented.

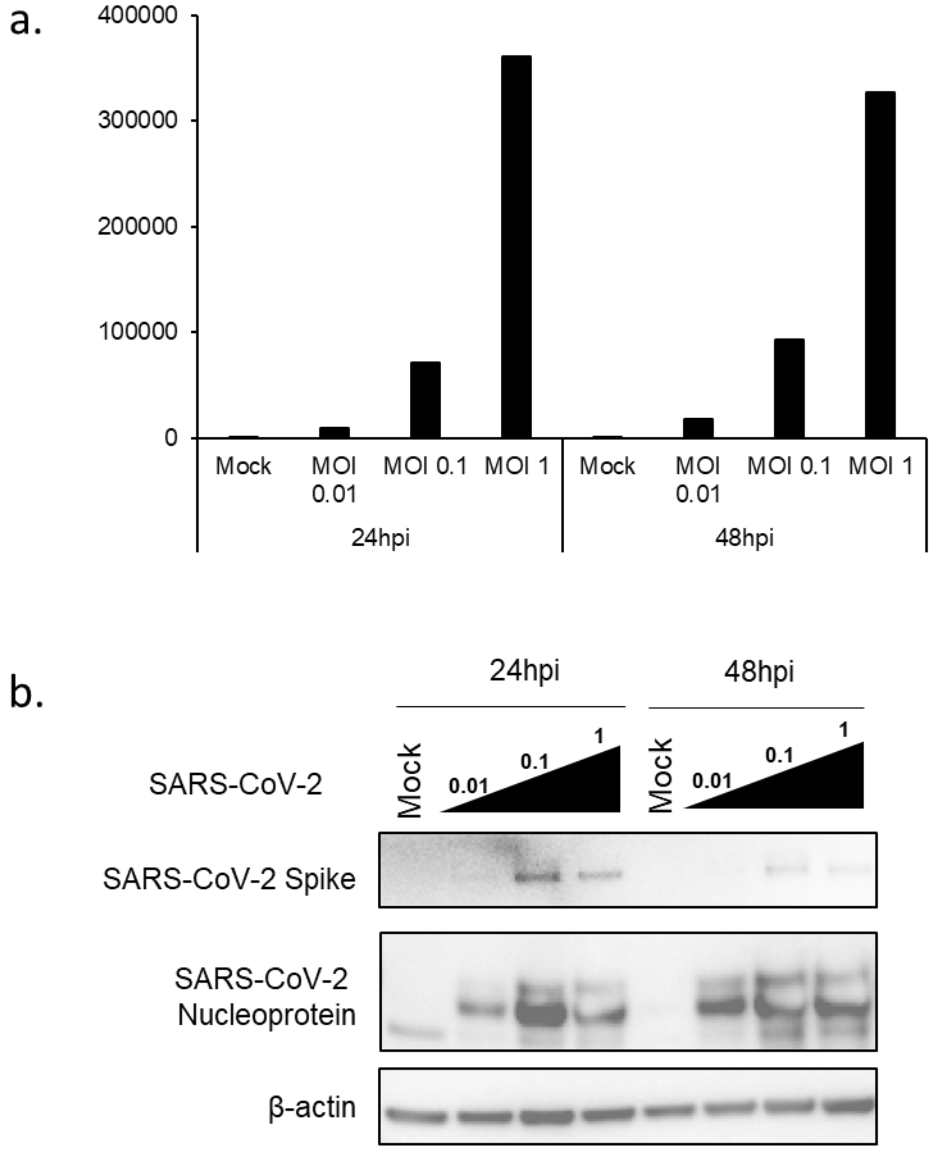

Figure S3 - (a) The viral RNA quantification using qPCR targeting the E gene of SARS-CoV-2 targeting the supernatant at 24hpi and 48hpi in Huh7 cell. The relative fold change with respect to the uninfected control is shown. (b) Western blot showing SARS-CoV-2 spike protein and Nucleoprotein at 24hpi and 48hpi in Huh7 cell.

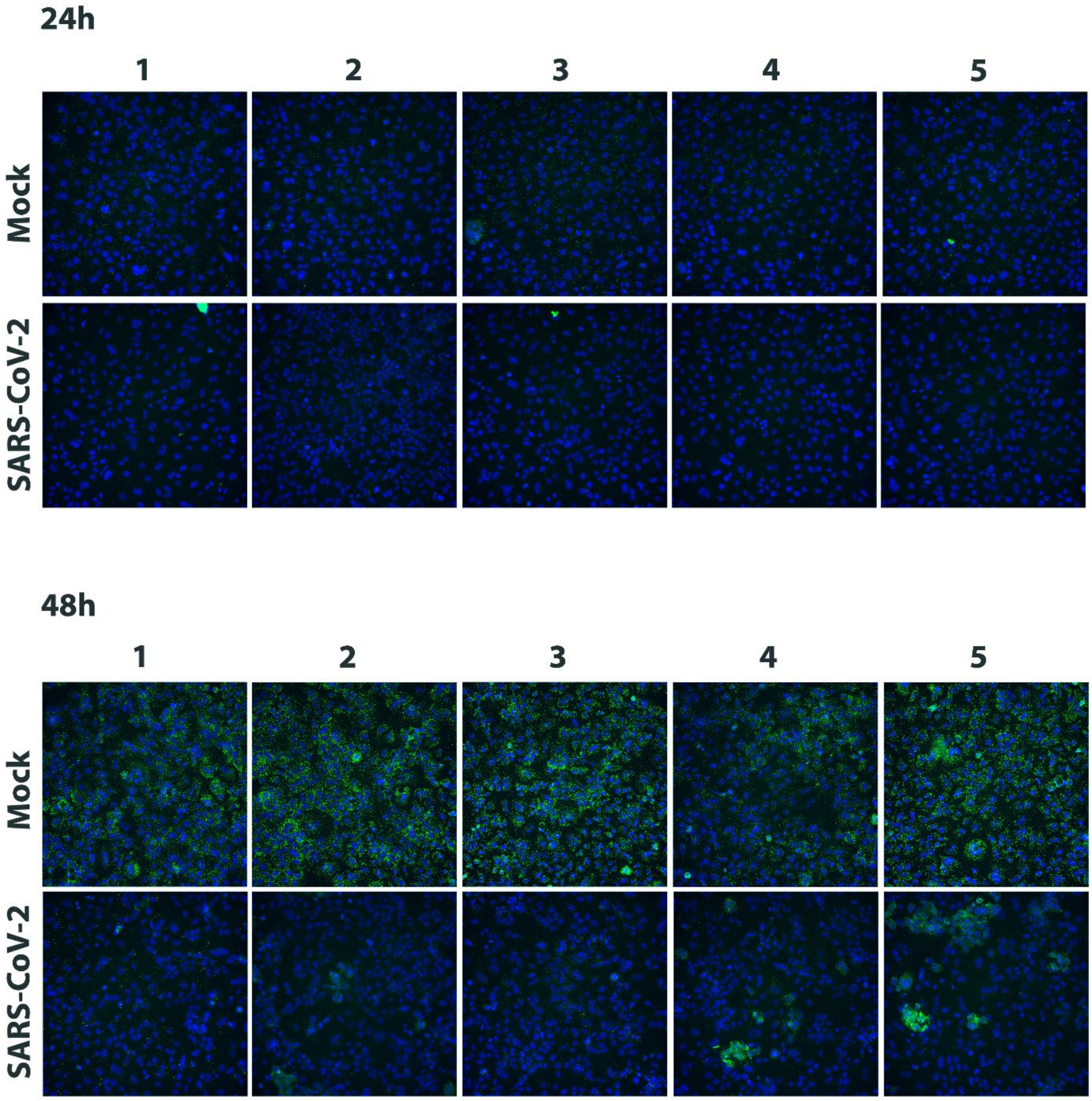

Figure S4. RNAScope® targeting HIF-1α (in green) captured in different microscopic fields.

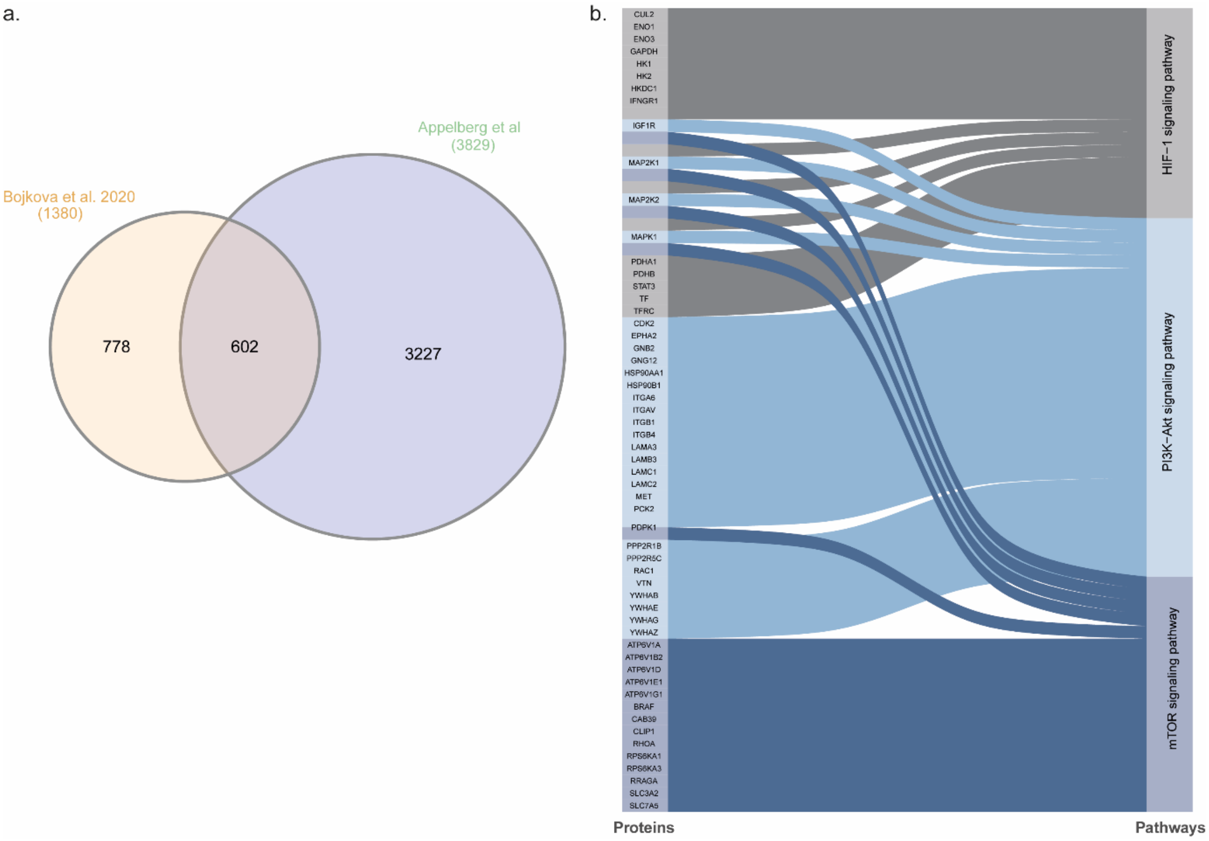

Figure S5. Overlap between differential protein abundance in Caco-2 (as reported by Bojkova et al 2020) and Huh-7. (a) Among the proteins 602 differentially abundant in both the cell lines. (b) Several proteins are part of HIF-1, mTOR and PI3K-Akt signaling pathway.
